# Assessing Putative Markers of Colorectal Cancer Stem Cells: From Colonoscopy to Gene Expression Profiling

**DOI:** 10.3390/diagnostics12102280

**Published:** 2022-09-21

**Authors:** Irina Florina Cherciu Harbiyeli, Daniela Elena Burtea, Elena Tatiana Ivan, Ioana Streață, Elena Raluca Nicoli, Daniel Uscatu, Mircea-Sebastian Șerbănescu, Mihai Ioana, Peter Vilmann, Adrian Săftoiu

**Affiliations:** 1 Research Center of Gastroenterology and Hepatology Craiova, University of Medicine and Pharmacy Craiova, 200349 Craiova, Romania; 2 Human Genomics Laboratory, University of Medicine and Pharmacy of Craiova, 200349 Craiova, Romania; 3 Department of Medical Informatics and Biostatistics, University of Medicine and Pharmacy of Craiova, 200349 Craiova, Romania; 4 Gastro Unit, Division of Endoscopy, Copenhagen University Hospital Herlev, 2730 Herlev, Denmark

**Keywords:** colorectal cancer, putative cancer stem cells, genetic biopsies

## Abstract

Cancer stem cells (CSCs) are proposed to be involved in colorectal cancer (CRC) initiation, growth, and metastasis. The aim of our pilot study was to assess possible correlations between the clinicopathological characteristics of CRC patients and CSCs gene expression patterns, in order to provide insight into new methods for patient stratification and targeted therapeutic strategies. Our study involved 60 CRC patients, and the following three specific CSC genes were targeted: PROM1/CD133, ALCAM/CD166 and HCAM /CD44. Data are presented as relative mRNA expression of target genes to GAPDH. The expression of total CD133 and CD166 was assessed in paired samples of CRC tumors and adjacent tissue, while CD44 was assessed in similar samples. The qRT-PCR analysis detected all three targeted genes to different extents, in both normal and tumor tissue. In nine cases (15.69%), total CD133 had a higher expression in tumor tissue, whilst in 28 cases (47.06%) the expression was higher in non-malignant peritumor tissue. The total CD166 expression was increased in tumor tissue compared with paired non-invaded peritumor samples in eight cases (13.73%), whilst in eight cases (13.73%) the expression was higher in non-malignant peritumor tissue. Total CD44 expression was higher in tumor tissue compared with paired non-invaded peritumor samples in 47 cases (78.95%). In the remaining cases the difference between paired samples was biologically insignificant. In conclusion, our study suggests that qRT-PCR is feasible in assessing the gene expression profiles of CSCs from CRC, and a promising pathway to be followed for determining how often a person needs screening by colonoscopy and at which age to start. This could improve CRC diagnosis and early patient stratification, and open the way for new oncologic treatment development.

## 1. Introduction

Colorectal cancer (CRC) is an environmental and genetic disorder. It is one of the most aggressive cancers worldwide, while tumor progression and metastasis constitute the primary cause of death. Cancer is defined by a great diversity of factors including gene expression, differentiation phenotypes, and tumor-host interactions [1]. The cell cycle involves distinct phases, being a complex and rigorously controlled process. Cell cycle regulation is conditioned by phase-specific transcriptions of cell cycle genes. Normal cells might be susceptible to have a cancerous phenotype as a consequence of altered cell cycle genes. The traditional concept regarding CRC carcinogenesis consists of several events, such as activation of oncogenes accompanied by the inactivation of tumor suppressor genes. Essentially, the genetic mutation of any mature colorectal cells is followed by uncontrolled cell differentiation within the tumor microenvironment and a consequent potential to invade the rest of the body [1,2]. On the other hand, considering the fast turnover of intestinal epithelial cells whose existence is too short to accumulate enough genetic alterations to generate tumors, it has been thought that CRC might derive from intestinal long-living stem cells [3]. Stem cells residing in the colon crypts are suggested to be the origin of both colon mature cells and CRC cells. The CRC stem cells theory states that tumors are organized hierarchically, with only the self-renewal subpopulation of cancer stem cells (CSCs) carrying the responsibility for tumor initiation, development, maintenance, metastasis, and treatment failure. However, the deregulation of the cell cycle progression in CSCs still remains incompletely understood [2,4,5].

Considering all these important features, CSCs emerge as a compelling topic for clinical and basic science studies, being essential to describe the expression of key genes as diagnostic markers for specific CSCs.

Previous studies have shown that CRC cells expressing high levels of CD133, CD166 or/and CD44 are different from the other bulk correspondents in their functions, morphology, and genomics. Dissimilarities were observed even between cells expressing moderate versus high levels of CD133, CD166 or/and CD44, in the way that cells with a high combined expression carried stem cells features like self-renewal in vivo and in vitro or the capacity to generate various cell phenotypes, while the moderate pattern cells were lacking these characteristics. The expression of CSCs could vary due to diverse factors, among which gene mutations must be noted [2,6,7,8].

Initially, the CD133 glycoprotein was used as an antibody targeting the AC133 epitope, in order to recognize colon CSCs. Currently, the literature proposes several other additional cell surface markers, such as CD44, CD166, beta 1 integrin- CD29, Lgr5, CD24, aldehyde dehydrogenase 1, EpCAM, DCAMLK1, Msi-1, EphB, and leucine-rich repeat-containing G-protein-coupled receptor 5 [3,9,10]. The CD133 gene is located on chromosome 4p15.32, it contains 37 exons and spans up to 160 kb. It is also known as Prominin-1, a cell surface glycoprotein composed of five transmembrane regions and two extracellular loops, with a molecular weight of 97–120 kDa [11]. Several studies have shown the increased tumorigenic potential of CD133 positive CRC cells [12,13], its elevated expression being correlated with chemo–radiotherapy resistance [12,14,15], distance metastasis and poor prognosis [16,17].

The CD166 gene is located on chromosome 3q13.1, it contains 16 exons and spans nearly 150 kb [18]. It is also known as the activated leukocyte cell adhesion molecule (ALCAM), a member of a subfamily of immunoglobulin receptors containing five extracellular domains. It is responsible for cell adhesion and migration. Furthermore, CD166 is correlated with reduced survival and CRC progression [19,20].

The CD44 gene is located on chromosome 11.p13, it is composed of 20 exons spanning a length of 60 kb. The protein encoded by this gene (also known as HCAM) is a cell-surface glycoprotein, a receptor for hyaluronan, which serves important roles in regulating cell adhesion, proliferation, growth, survival, motility, migration, angiogenesis, and differentiation [21,22,23,24]. CD44 has implications in tumor-progressing function, leading to tumor metastasis with poor prognosis [25,26].

CD133, CD166 and CD44 are described as useful markers for the isolation and supplementary characterization of colorectal CSCs [16]. However, the interconnections between the expression of CSCs markers, CRC metastasis and clinico-pathological features are still not fully explained. Moreover, there are limited studies targeting mRNA expression of CSCs while utilizing confirmed and practical procedures such as reverse transcription polymerase chain reaction (RT-PCR).

The promise of the CSCs hypothesis is that a rigorous understanding of CSC biology will allow the development of more effective procedures to eradicate CSCs in CRC patients. Inhibition of key CSC signaling pathways, viral therapy, awakening quiescent CSCs, and immunotherapy represent some of the strategies that have been tested to disrupt the CSCs trajectory [26]. Other novel cancer treatment methods, such as application of the decoy oligodeoxynucleotides (ODNs) strategy in colon cancer stem cell elimination, could boost the sensitivity of cancer cells to irradiation. In consequence, the eradication of cancerous cells from tumors will support cancer treatment. Another possible therapy approach, in addition to conventional cancer treatment, might target suppression of Oct4 and Sox2 transcription factors, as it is known that Oct4–Sox2 decoy ODNs can induce apoptosis, decrease proliferation, and inhibit migration, invasion, and colony formation ability [27,28].

Taking into consideration the increasing evidence regarding the critical role that CSCs play in both tumor initiation and tumor resistance and relapse following chemo–radiotherapy, the aim of the present pilot study was to investigate possible correlations between the clinicopathological characteristics of CRC patients and the CD133, CD166, and CD44 gene expression patterns, in order to provide insight into additional methods for patient stratification, followed by the development of targeted therapeutic strategies.

The novelty of our study consists of the simultaneous assessment of the CD133, CD166, and CD44 gene expression patterns in both tumor and normal samples of CRC patients, obtained through minimally invasive colonoscopy procedures. Previous genetic studies have focused on individual CSCs markers, rather than the association of the three markers included in our study, or their expression characteristics were determined only in post-surgical tumor tissues.

## 2. Materials and Methods

### 2.1. Patients and Specimens

Our study involved 60 CRC patients who were subjected to qRT-PCR assessment of the following three specific CSCs gene expressions: PROM1/CD133, ALCAM/CD166, and HCAM/CD44. Fresh tumor and peritumor biopsies were harvested one by one during colonoscopy, at the Research Centre in Gastroenterology and Hepatology of Craiova, Romania. The peritumor samples were independently obtained after performing a colonoscopic biopsy of normal-appearing mucosa, adjacent to the colorectal tumor. All specimens were collected in an RNA stabilization solution (RNAlater, Ambion, Inc., Austin, Texas, US) and stored at −80° until mRNA was extracted at the Human Genomics Laboratory, University of Medicine and Pharmacy of Craiova.

Additional tumor and peritumor specimens were collected during colonoscopy procedures and stored in formalin for pathological examination. None of the biopsied peritumor samples showed macroscopic signs of malignancy. This was confirmed by histopathological examination, which revealed that peritumor tissues were not microscopically invaded by malignant cells.

None of the patients had received either chemotherapy or radiation therapy prior to the sample collection.

The study was reviewed and approved by the University of Medicine and Pharmacy of Craiova Institutional Review Board.

All study participants provided informed written consent prior to study enrollment.

### 2.2. mRNA Extraction

RNA was isolated and purified using the PureLink^®^ RNA Mini Kit from Invitrogen (Life Technologies, Carlsbad, CA, USA).

### 2.3. Assessment of RNA Concentration, Purity and Degradation

Sample quality was assessed by agarose gel electrophoresis and spectrophotometry (A260/A280 ratio) using aliquots of total RNA, to evaluate whether the RNA was of sufficient quality to continue. If the total RNA appeared intact, the samples were prepared for reverse transcription. All 60 samples collected met quality criteria. The RNA concentration and purity were measured spectrophotometrically (Eppendorf Biophotometer, Eppendorf, AG, Hamburg, Germany)). Spectrophotometrically, measurements at 260 nm for RNA concentration should be greater than 0.15. An absorbance of 1 unit at 260 nm corresponds to 44 µg of RNA per ml, at a neutral pH. From the relative absorbance at 230, 260 and 280 nm (i.e., A260/A280 and A260/A230) by spectrophotometry, RNA purity can be estimated. The ratio between the absorbance values at 260 and 280 nm represents the RNA purity with respect to proteins. The ratio between the absorbance values at 260 and 230 indicates the contamination with guanidine thiocyanate. A pure RNA sample should have an A260/A280 ratio of 1.7–2.1 and A260/A230 ratio of 1.8–2.2. The RNA concentration of the samples varied between 400 and 1000 µg/mL. All RNA samples were brought to a concentration of 100 ng/µL before reverse transcription. All of the 260/280 and 260/230 ratios were within recommended ranges for use in reverse transcription.

### 2.4. Two Step qRT-PCR

qRT-PCR is the gold standard technique for measuring gene expression, enabling the detection of differences of only a few copies of mRNA per cell. The analysis of gene expression using real-time PCR provides the opportunity to quantify the different expression levels of a given gene in a patient population. To be able to perform quantitative real-time PCR, in vitro synthesis of the complementary strand from the mRNA is required (reverse transcription—RT). Once the cDNA has been synthesized, quantitative real-time PCR can be performed. The reverse transcription step is the main variable of this reaction. To overcome this obstacle and to obtain reliable results we used a two-step RT-PCR assay: the RT reaction and real time PCR amplification. The first step is the synthesis of complementary DNA (cDNA) by reverse-transcription. In a second step, PCR products are synthesized and evaluated quantitatively from cDNA using TaqMan technology.

### 2.5. Reverse-Transcription

The reverse-transcription was performed using a High Capacity cDNA Reverse Transcription Kit (Applied Biosystems, Foster City, CA, USA). The reverse transcription reactions were carried out in 20 µL volume; the input amount of total RNA was 1 µg diluted to a volume of 10 µL in nuclease-free water. A 2X Reverse-Transcription Master Mix was prepared according to Table 1 (Applied Biosystems, Foster City, CA, USA).

The reactions were carried out in a thermal cycler (Eppendorf Mastercycler) using the cycling conditions described in Table 2.

### 2.6. Quantitative Real-Time Polymerase Chain Reaction (qRT-PCR)

In the second step, the PCR products were amplified and quantified using the TaqMan^®^ Gene Expression Master Mix and specific TaqMan Gene Expression Assays (Applied Biosystems, Foster City, CA, US). The TaqMan^®^ Gene Expression Master Mix contains AmpliTaq Gold^®^ DNA polymerase, UP (UltraPure), uracil-DNA glycosylase (UDG), deoxyribonucleotide triphosphates (dNTPs) with deoxyuridine triphosphate (dUTP), ROXTM passive reference, and buffer components. AmpliTaq Gold^®^ DNA polymerase, UP, a chemically modified form of AmpliTaq^®^ DNA polymerase, is an essential ingredient in hot start PCR. The thermal incubation step required for activation ensures that active enzyme is generated only at temperatures where the DNA is fully denatured.

The AmpliTaqGold^®^ DNA polymerase, UP enzyme is identical to AmpliTaq Gold DNA polymerase, but further purified in order to reduce bacterial DNA introduced from the host organism. The purification process ensures that non-specific, false-positive DNA products due to bacterial DNA contamination are minimized during PCR. Uracil-DNA glycosylase (UDG, also known as uracil-N-glycosylase (UNG)) treatment can prevent the re-amplification of carryover-PCR products by removing any uracil incorporated into single- or double- stranded amplicons.

The ROXTM passive reference provides an internal reference to which the reporter dye signal can be normalized during data analysis. Normalization is necessary to correct for fluorescent fluctuations due to changes in concentration or volume.

The TaqMan^®^ MGB probes consist of a target-specific oligonucleotide with: a reporter dye (for example, 6FAMTM dye) linked to the 5′ end of the probe; a minor groove binder (MGB), which increases the melting temperature (Tm) without increasing probe length; and a nonfluorescent quencher (NFQ) at the 3′ end of the probe, which offers the advantage of a lower background signal, resulting in better precision quantitation.

The PCR reaction exploits the 5′ nuclease activity of AmpliTaq^®^ Gold DNA polymerase, UP (UltraPure) to cleave a TaqMan^®^ probe during PCR. During the reaction, cleavage of the probe separates the reporter dye and the quencher dye, resulting in increased fluorescence of the reporter. Accumulation of PCR products is detected directly by monitoring the increase in fluorescence of the reporter dye. When the probe is intact, the proximity of the reporter dye to the quencher dye results in suppression of the reporter fluorescence, primarily by Förster-type energy transfer. During PCR, if the target of interest is present, the probe specifically anneals to the target. The 5′ to 3′ nucleolytic activity of the AmpliTaq Gold, UP enzyme cleaves the probe between the reporter and the quencher only if the probe hybridizes to the target. Consequently, the fragments of the probe are dislocated from the target and polymerization of the strand continues. The 3′ end of the probe is blocked to prevent extension of the probe during PCR. This process occurs in every cycle, and it does not interfere with the exponential accumulation of product. The increase in fluorescence signal is detected only if the target sequence is complementary to the probe and if it is amplified during PCR. Because of these requirements, nonspecific amplification is not detected.

Normalization is accomplished by dividing the emission intensity of the reporter dye by the emission intensity of the ROX passive reference to obtain a ratio defined as the Rn (normalized reporter) for a given reaction tube. The threshold cycle (CT) value is the cycle at which a statistically significant increase in ΔRn is first detected. Threshold is defined as the average standard deviation of Rn for the early cycles, multiplied by an adjustable factor (Applied Biosystems, 2009; US). The amplifications were carried out in 20 µL volume, in triplicate. The cDNA was diluted 1:10 in nuclease-free water prior to use in the PCR reaction. The PCR reaction components are listed in Table 3.

The TaqMan Gene Expression Assays used are listed in Table 4. All of the probes were designed to span an exon–exon boundary, in order to avoid unspecific amplification of residual genomic DNA. The expression of the target genes was normalized to the GAPDH endogenous control gene.

### 2.7. Statistical Analysis

Comparative expression of targeted genes in paired tumor and peritumor mucosa was assessed by the Wilcoxon matched pairs signed rank test. Data are presented as relative mRNA expression of the target gene to GAPDH (as a housekeeping gene). Results were considered statistically significant when *p* < 0.0001.

## 3. Results

A total of 60 patients diagnosed with CRC were investigated using qRT-PCR for the assessment of three specific CSC gene expression levels: PROM1/CD133, ALCAM/CD166, and HCAM/CD44. We observed a higher proportion of men versus women, living in urban areas, with an age varying between 25 and 80 years old, 53 patients being over and seven patients under 50 years old. Most patients had advanced tumors (T1/T2—8 cases and T3/T4—52 cases), whilst 36 patients presented nodal involvement. The overall survival 3 years post-diagnosis was 70%. Patients’ clinical and pathological data are presented in Table 5.

To investigate CD133, CD166, and CD44 mRNA expression profiles, relative mRNA levels (target gene/GAPDH) were assessed in all the samples. Relative mRNA levels for each of the lesions included were compared with the relative expression in normal colorectal mucosa. The qRT-PCR analysis of the paired biopsies detected all three genes to different extents, in both normal mucosa and also in the tumor tissue.

Concerning the degree of gene expression levels, several annotations were applied throughout the text: T—when higher expression levels were found in tumor, N—higher expression levels were found in normal tissue, I—when the differences between normal and tumor tissues were insignificant.

**Figure 1:**
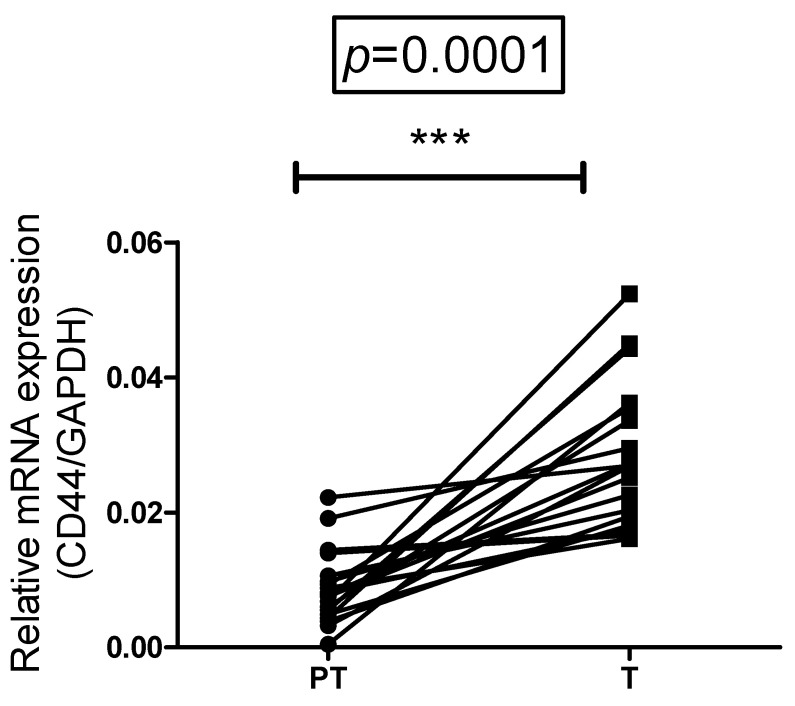
Comparative expression of CD44 mRNA in paired tumor and peritumor mucosa. Data are presented as relative mRNA expression of target gene to GAPDH. Wilcoxon matched pairs signed rank test, *p* < 0.0001. *** The difference between the matched pairs is statistical significant.

The expression of total CD44 (HCAM) was assessed in paired samples of CRC tumors and adjacent tissue. qRT-PCR analysis revealed that CD44 is expressed in both tumor and peritumor mucosa. Total CD44 expression was higher in tumor tissue, compared with paired non-invaded peritumor samples in 78.95% of cases, whilst in the remaining 21.05% the difference between paired samples was biologically insignificant (Figure 1).

**Figure 2:**
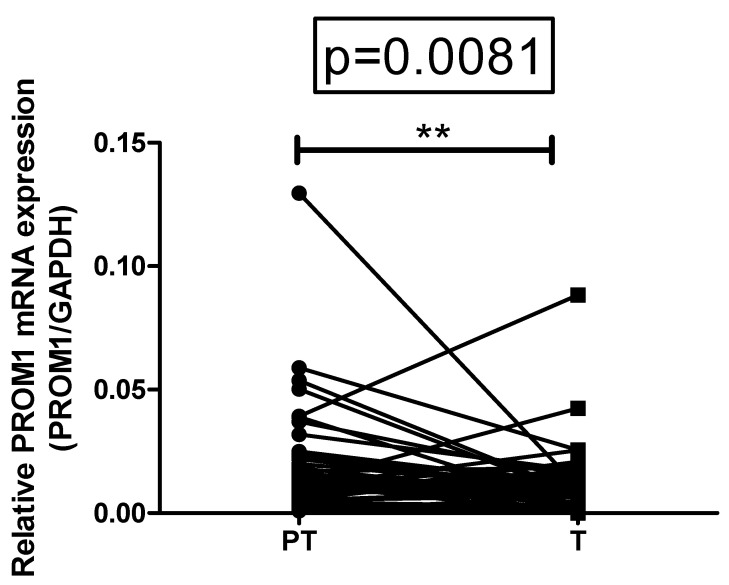
Comparative expression of PROM1 mRNA in paired tumor and peritumor mucosa. Data are presented as relative mRNA expression of target gene to GAPDH. Wilcoxon matched pairs signed rank test, *p* < 0.0001. ** The difference between the matched pairs is statistical significant.

The expression of total CD133 (PROM1) was assessed in paired samples of CRC tumors and adjacent tissue. Total CD133 expression was higher in tumor tissue, compared with paired non-invaded peritumor samples in 15.69%; in 47.06%, the expression was higher in non-malignant peritumor tissue, and in the remaining 37.25% the difference between paired samples was biologically insignificant (Figure 2).

**Figure 3:**
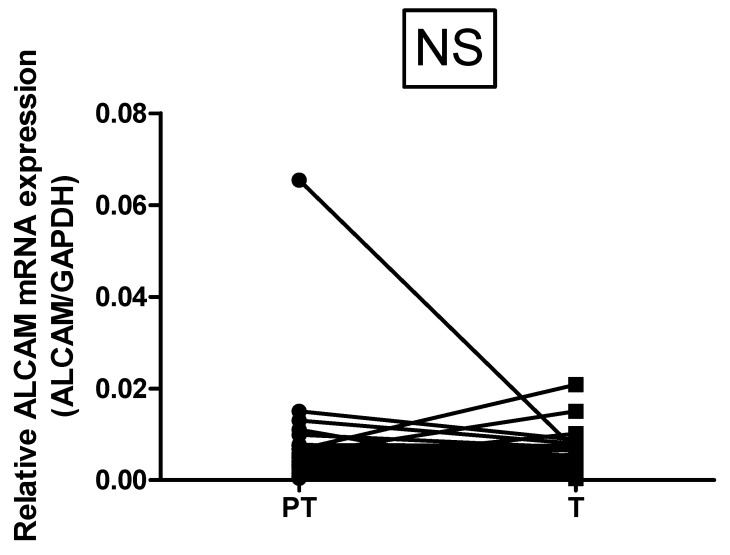
Comparative expression of ALCAM mRNA in paired tumor and peritumor mucosa (n = 51). Data are presented as relative mRNA expression of target gene to GAPDH. Wilcoxon matched pairs signed rank test, *p* < 0.0001.

The expression of total CD166 (ALCAM) was assessed in paired samples of CRC tumors and adjacent tissue. The total CD166 expression was increased in tumor tissue, compared with paired non-invaded peritumor samples in 13.73%; in 13.73%, the expression was higher in non-malignant peritumor tissue, and in the remaining 72.55% the difference between paired samples was biologically insignificant (Figure 3).

**Figure 4:**
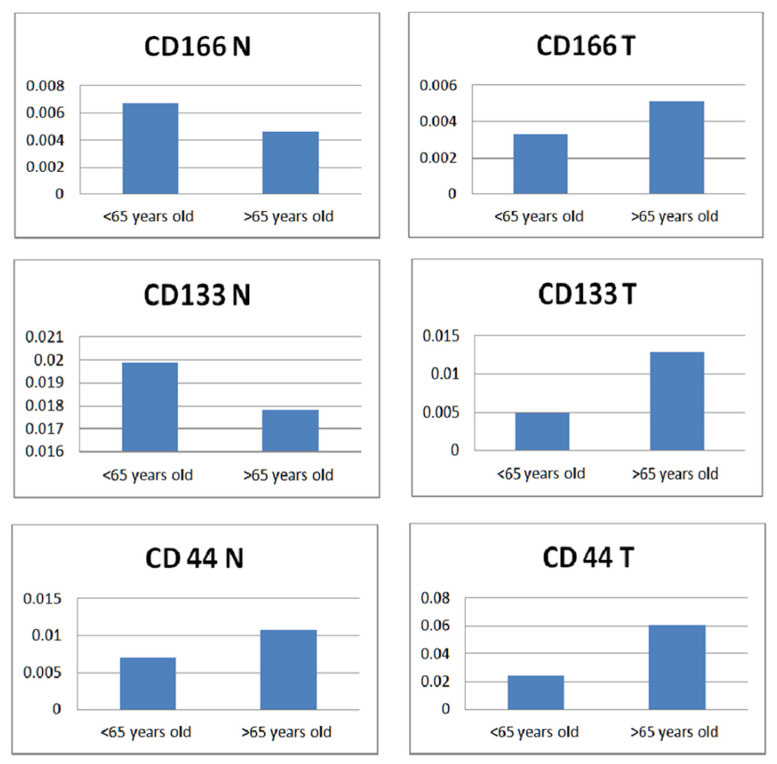
Normal versus tumor tissue expression of the three markers according to age. A non-parametric Mann–Whitney test was used to compare individual groups, and all values achieved statistical significance (*p* < 0.05).

The relationship of CD166 vs CD133 vs CD44 with age (Figure 4) revealed a similar higher expression of CD166 and CD133 in the normal tissue of patients under 65 and in the tumor tissue of the patients over 65 years old. All three markers were over-expressed in the tumor tissue of patients over 65 years old. When superimposing normal vs tumor tissues among each marker, only CD44 followed the same pattern in both tissues, with higher expression in patients over 65 years old, while the expression of CD166 and CD133 was completely opposite, each marker following the pattern of a mirror image.

**Figure 5:**
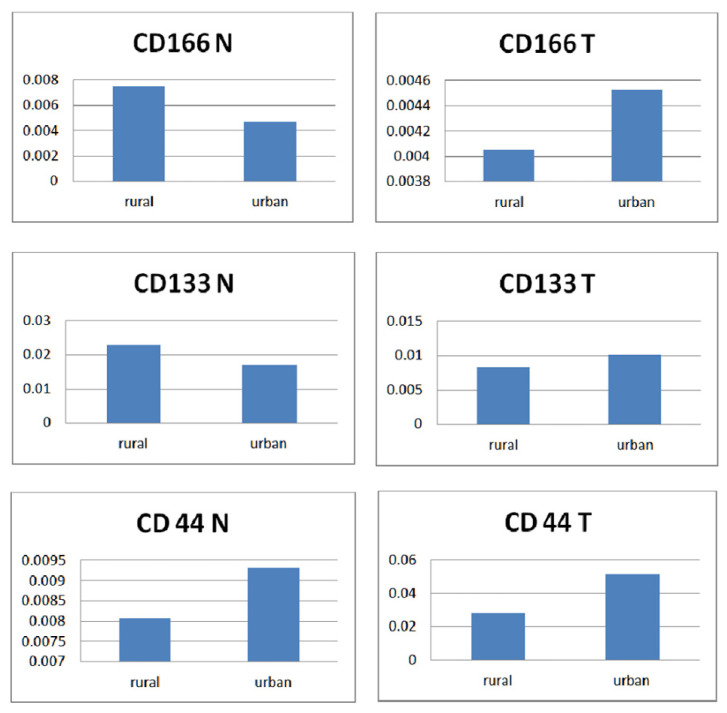
Normal versus tumor tissue expression of the three markers according to home location. A non-parametric Mann–Whitney test was used to compare individual groups, and all values achieved statistical significance (*p* < 0.05).

When correlating CD166 vs CD133 vs CD44 with home location (Figure 5) we noticed a similar higher expression of CD166 and CD133 in normal tissue among patients from rural areas. All three markers were over-expressed in the tumor tissue of patients originating in urban areas. When matching normal vs tumor tissues among each marker, only CD44 was dominantly expressed in patients from urban areas, regardless of the type of tissue, while the expression of CD166 and CD133 followed the pattern of a mirror image.

**Figure 6:**
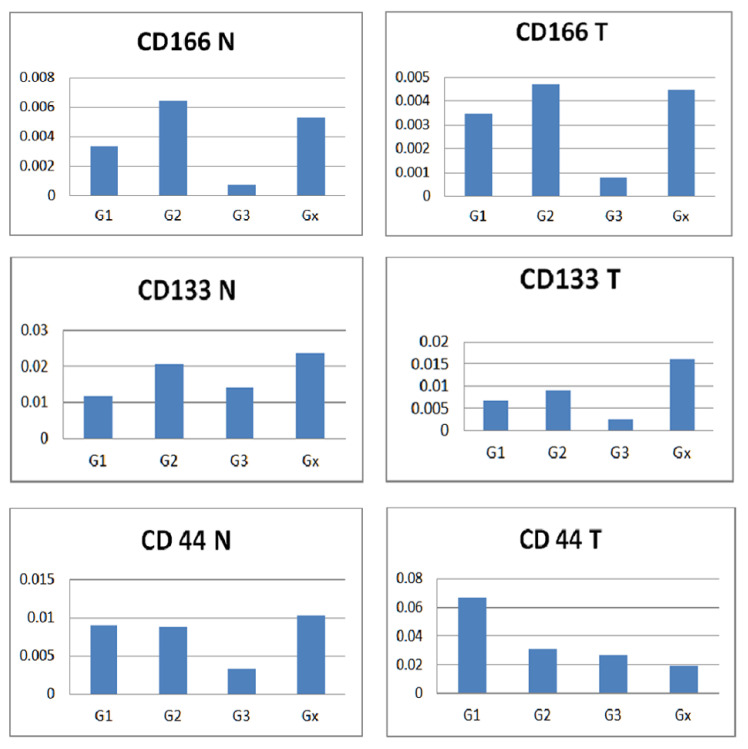
Normal versus tumor tissue expression of the three markers according to tumor grading. A non-parametric Kruskal–Wallis test was used to compare individual groups, and all values achieved statistical significance (*p* < 0.05).

Comparing CD166 vs CD133 vs CD44 expression patterns with the tumor grading (Figure 6), we observed the lowest levels of all three markers in normal tissue, grade G3, similarly in the case of CD166 and CD133 tumor tissue. When comparing normal vs tumor tissue for each marker, the highest expression of CD166 was related to G2 and the lowest to G3, while CD133 was associated with Gx. The only observation related to CD44 is the decreasing expression values from G1 to Gx.

**Figure 7:**
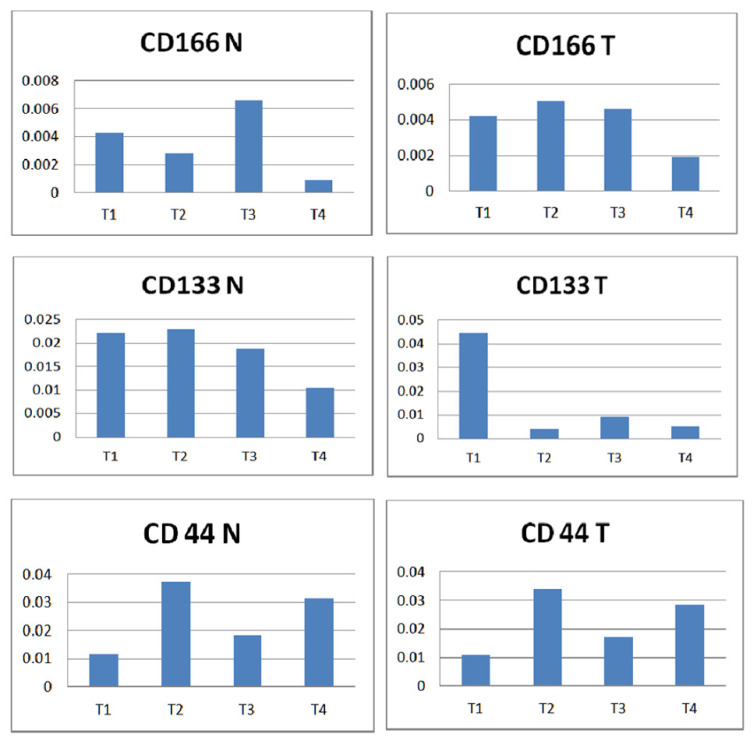
Normal versus tumor tissue expression of the three markers according to T staging. A non-parametric Kruskal–Wallis test was used to compare individual groups, and all values achieved statistical significance (*p* < 0.05).

When comparing CD166 vs CD133 vs CD44 according to tumor staging (Figure 7), only one pattern of expression was observed in the case of CD166 and CD44 that reached the highest levels in tumor tissue at the T2 stage. A considerably high expression of CD133 was noted in tumor tissue at the T1 stage. When comparing normal vs tumor among each marker, a similarity was noted with the lowest level of CD44 expression at T1, highest at T2, and T4 was higher than T3. In the case of CD166, the lowest expression was registered at the T4 stage, in both normal and tumor tissue.

**Figure 8:**
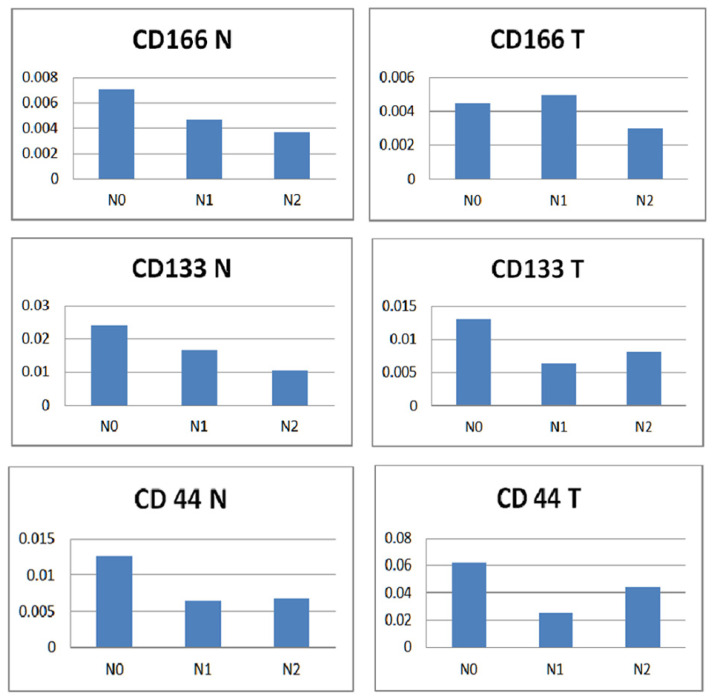
Normal versus tumor tissue expression of the three markers according to N staging. A non-parametric Kruskal–Wallis test was used to compare individual groups, and all values achieved statistical significance (*p* < 0.05).

Regarding the expression of N0, N1 and N2 levels (Figure 8) when comparing normal versus tumor tissue among each marker only, no patterns or associations were observed when comparing the charts. When comparing CD166 vs CD133 vs CD44, the highest expression levels of all three markers were observed in the normal tissue (stage N0). Only one exception was noted, which was the expression of CD166 in tumor tissue, where the highest level was associated with the N1 stage. A similar pattern of expression of both CD133 and CD166 was noted in the normal tissue, with decreasing values from N0 to N1 to N2. Another similarity was noticed when comparing the expression of CD133 and CD44 in tumor tissue, with the highest levels related to the N0 stage, which decreased in the N1 stage and increased in the N2 stage.

## 4. Discussion

CRC is a heterogeneous genetic and epigenetic disease, and the possible usage of DNA, RNA and various biomarkers for CRC characterization has been investigated during recent years. At the present time, there is no satisfactory evidence for introducing it into daily clinical practice, but the fast advances recorded in molecular biology make it a promising field [29].

Firstly formulated in 2007, a new theory regarding cancer pathogenesis, the CSCs hypothesis states that only a small proportion of tumor cells are capable of tumor initiation and development [30]. While the exact origin of CSCs remains unclear, two potential CSCs sources have been suggested: either normal stem cells or differentiated tumor cells. It is considered that CSCs acquire various genetic mutations leading to a modified gene expression array in CSCs, concurrently with tumorigenesis [31]. An important goal of cancer research is to establish the specific mechanisms associated with the appearance, uncontrolled self-renewal, dissemination and treatment resistance of CSCs [30,32].

Identifying and understanding CSCs will increase cancer diagnosis methods and enhance early patient stratification, leading to new and more efficient therapies specifically targeting this fraction of tumor cells and the improvement of the overall outcome of CRC patients [33,34]. As a consequence, we combined clinical settings with fundamental research, in order to assess genetic methods of identifying and characterizing CSCs.

Currently, qRT-PCR is considered the gold standard technique for measuring gene expression, enabling the detection of differences of only a few copies of mRNA per cell. Gene analysis using RT-PCR provides the opportunity to quantify different expression levels of a given gene in a patient population, thus validating imaging and other paraclinical findings at the molecular level [14].

Three specific CSCs genes were targeted in our study: PROM1/CD133, ALCAM/CD166, and HCAM/CD44, all of which have been established by several other studies involving IHC or molecular biology techniques. Thus, the combined use of biomarkers might be efficient to identify CSCs and tumors with a negative prognosis. Similar to the published findings, qRT-PCR analysis of paired biopsies detected all three genes to different extents, in both normal mucosa and tumor tissue [16,29,35].

In our study, CD44 expression was higher in tumor tissue, in agreement with previous published data [36]. On the other hand, CD133 had a higher activation level in the normal mucosa, which is opposite to some of the results mentioned in the literature. Therefore, while CD133 expression is described as heterogeneous in different types of tissues without statistical relevance [37], other studies claim an elevated expression level of CD133 in tumor tissue [38,39]. Regardless the inconsistent expression level of CD133 in different tissue types, the usage of qRT-PCR method led to statistically significant results. Hence, qRT-PCR carries a high potential for assessing CSCs expression levels.

A previous CRC study correlated the mRNA expression of CD44 and CD133 with the existence of synchronous hepatic metastases, revealing that the genes’ expression levels were highly co-expressed and decreased from hepatic metastasis tissue to CRC tissue, both preceding normal mucosa. Other clinicopathological parameters analyzed in the study were patient survival, tumor location, and histology according to mRNA expression, and it was noted that a high CD44 level in hepatic metastases was associated with a lower survival rate than in patients with low expression levels and all the other factors. On the other hand, CD133 was not associated with patient survival as an independent marker [37]. Another study found that colonospheres, obtained from either cells with high expression of CD133 or from CD133 negative cells after being magnetically separated, have totally distinct gene expression patterns [40].

The difference between the expression level of CD166 in normal mucosa and tumoral tissue was biological insignificant in the majority of the cases, although previous papers have described it as being present predominantly in tumor cells. Regarding CD166 and CD44, a RT-PCR analysis of normal mucosa and adenomatous polyps revealed increasing expression levels of both markers in parallel with advancing patient age, hence an age-related enlarging of the CSCs population. Moreover, it was observed than subjects over 55 years old had a higher expression of the two co-expressed markers in normal mucosa than patients under 55 years old and diagnosed with adenomas. In essence, the proportion of stem cells widens in parallel with aging, hence the higher susceptibility of colorectal mucosa to undergo several alterations with time [41].

Approximately 83% of current CSC surface markers appear to be present on human embryonic stem cells or adult tissue stem cells, the population being scarcely expressed on normal tissue cells. The biomarker CD133 is rarely expressed on normal tissue cells, while CD166 and CD44 are expressed on both adult stem cells and normal tissues (epithelial or lymphatic tissues). In our study, CD133, CD166, and CD44 expression was found in samples of histologically normal non-neoplastic colorectal mucosa. The different observed patterns could be attributed to expressions of different CD44 isoforms (splice variants), and it is possible that the peritumor tissues had been subjected to changes at the molecular level. To overcome the ambiguity of these CSCs markers, a control group of non-oncologic subjects would be desirable, and CSCs-specific epitopes are necessary to analyze functional CSC activity [42,43].

Quantification of gene expression has the potential to divulge important knowledge regarding the mechanisms developed within a cell. The activation or repression status of a gene illustrates its altered functions and the disturbed pathways which lie at the base of cells’ individuality and behavior. Although RT-PCR has the potential to provide evidence regarding the expression of numerous genes at once, it is still necessary to validate the results through other procedures, especially when RNA amplification is necessary due to the limited quantity of RNA available [44]. The lack of usage of other CSCs investigation methods might represent a theoretical limitation of the present study, although we have previously assessed the expression patterns of CSCs biomarkers (CD133 and CD166) in an enzymatic and multiple fluorescence immunohistochemistry study. In this study, we observed a direct correlation between CD133 and CD166 expression levels throughout the entire spectrum of lesions and that CD133/CD166 colocalization is an early event occurring in colon tumorigenesis, with the highest coefficients recorded for patients with high grade dysplasia, followed by well-differentiated tumors. In consequence, we consider that the co-expression of these two markers could be useful for further prognostic and therapeutic stratification of patients with colon cancer [45]. Another study that analyzed large-scale recognized IHC markers of CRC highlighted that CD44v6 can be used to predict the position of tumors bigger than 5 cm and is also a predictive marker for poor disease-free survival in CRC. The overexpression subgroup associated with poor overall survival contained both CD44v6 and CD133 antigens. Due to the selection criteria, CD166 was not included in this meta-analysis [46]. In this regard, the clinico-pathological significance of our data partially correlates with the findings of the two studies mentioned above.

Clinical trials have emphasized the need for molecular stratification parameters to select the subset of high-risk patients with early-stage CRC who would benefit from adjuvant therapy, hence for recurrence/progression risk stratification, more aggressive treatment decisions, and hopefully improved patient prognosis [47,48]. Going further than the actual focus on optical colonoscopy and histopathological evaluation of resected polyps, the GENESIS study empowers the idea of genetic biopsy, revealing dissimilar mutational landscapes according to polyp size and location [49]. Similarly, in our study we looked for new molecular stratification parameters, taking into consideration the importance of basic and applied science in gastroenterology. Our study confirmed that the assessment of genetic patterns can be included in the workflow of contemporary colonoscopy practice. We acknowledge sample size as a major limitation to the present study, together with the lack of inclusion of other CSCs investigation methods. Another drawback could be the short term nature of our study. A more extensive period of time would allow the dynamic comparison of CD133, CD166, and CD44 expression patterns before and after oncology treatment. Additionally, the lack of colorectal biopsies obtained from healthy subjects could be considered a limitation of our study, as this might properly reflect the expression of the non-neoplastic stem cells.

### Future Perspectives

Since formulating the concept of CSCs in 2007, major advancements in CSCs research have taken place during the recent years, due to the development of cellular and molecular technologies. Hence, the definition of CSCs expanded from a cell subpopulation with a stable phenotype to a plastic entity impacted by polyvalent interactions with the tumor microenvironment [31]. This distinctive population of cells, if left unchecked, leads to exacerbated tumorigenicity, metastases and recurrence. Therefore, it is essential to develop therapies that selectively target CSCs, in order to limit the toxicity of conventional chemo–radiation and improve patient outcomes. Regarding personalized medicine, a promising method of testing specific therapies is cultivating CSCs organoids [50]. CSC-targeted therapies address kinase inhibitors, or stem cell associated pathways such as Wnt and β-catenin. Several of these approaches have already entered the clinical phase [51,52]. Although gene therapy for CRC has made important advancements, more fundamental studies are required. Currently, the TP53 and KRAS genes are the two main players in CRC carcinogenesis that serve as valuable targets for gene therapy [53].

Several CSCs inhibitors have been tested in clinical trials as anti-CRC agents (buparlisib, napabucasin, curcumin, MK-2206, epigallocatechin-3-gallate, metformin, vismodegib, taselisib, quercetin, and rapamycin) but the data is still limited. Currently, there is no clinically effective therapy to suppress CSCs; however, it is expected that combining conventional oncotherapies with CSCs-targeting drugs [54], discovering each patient’s unique tumor-associated antigens and the development of personalized therapies, optimized in the future by more advanced technologies and bioinformatics methods, will lead to better cure rates, and improve prognosis, survival and quality of life for CRC patients [55].

## 5. Conclusions

Our study suggests that qRT-PCR is feasible in assessing the gene expression profiles of CSCs from CRC, and is a promising pathway to be followed for determining how often a person needs screening by colonoscopy and at which age to start, for improving CRC diagnosis, early patient stratification and opening the way for new oncologic treatment development. The combination of multiple targeting strategies should be considered for the development of CSCs-directed therapeutic strategies.

## Figures and Tables

**Table 1:** 2XRT Master Mix (for 1 reaction).

Component	Volume (µL)
10X RT Buffer	2
25X dNTP Mix (100 mM)	0.8
10X RT Random Primers	2
Multiscribe^TM^ Reverse Transcriptase	1
RNAase Inhibitors	1
Nuclease-Free dH_2_O	3.2
Total per Reaction	10.0

**Table 2:** Cycling parameters for Reverse Transcriptions.

	Step 1	Step 2	Step 3	Step 4
Temperature (°C)	25	37	85	4
Time	10 min	120 min	5 sec	∞

**Table 3:** Thermal cycling conditions for Real-Time PCR.

Step	UDG Incubation	AmpliTaq Gold, UP Enzyme Activation	PCR	
	HOLD	HOLD	CYCLE (50 Cycles)	
			Denature	Anneal/Extend
Time	2 min	10 min	15 sec	1 min
Temperature (°C)	50	95	95	60

**Table 4:** TaqMan Gene Expression Assays.

Gene	Transcript	Exon boundary	Amplicon size	Code
GAPDH	NM_002046.3	3-3	122	Hs99999905_m1
CD44	NM_001202556.1	7-8	70	Hs01075861_m1
CD166	NM_001243280.1	2-3	103	Hs00977641_m1
CD133	NM_001145850.1	6-7	66	Hs01009259_m1

**Table 5:** Patient characteristics.

**Mean age ± SD (range) (yrs)**	63.72 ± 11.16 (25–80)
**Gender (M/F)**	50/10
**Home location (Urban/Rural)**	43/17
**Tumor location** Descending colon Sigmoid Recto-sigmoid junction Rectum	2 6 3 49
**Histology** Adenocarcinoma G1 G2 G3 Undetermined	12 33 2 13
T stage * T1 T2 T3 T4	2 6 43 9
N stage * N0 N1 N2	24 24 12

T stage * refers to the size and the extent of the tumor; N stage * refers to the regional lymph nodes that are involved.

## Data Availability

All data supporting the study can be located in the archive of Research Center of Gastroenterology and Hepatology Craiova, Romania.
